# Highlighting the Potential for Chronic Stress to Minimize Therapeutic Responses to Radiotherapy through Increased Immunosuppression and Radiation Resistance

**DOI:** 10.3390/cancers12123853

**Published:** 2020-12-20

**Authors:** Minhui Chen, Anurag K. Singh, Elizabeth A. Repasky

**Affiliations:** 1 Department of Immunology, Roswell Park Comprehensive Cancer Center, Buffalo, NY 14263, USA; minhui.chen@roswellpark.org; 2 Department of Radiation Oncology, Roswell Park Comprehensive Cancer Center, Buffalo, NY 14263, USA; Anurag.Singh@roswellpark.org

**Keywords:** chronic stress, immunosuppression, adrenergic signaling, radiation therapy, abscopal effect

## Abstract

**Simple Summary:**

Stress is an integral part of life and is necessary for proper development and function of every organ. However, there is growing evidence that prolonged activation of the sympathetic stress response stress negatively affects the outcome of many diseases including cancer and impairs the efficacy of widely used therapies. In this review, we specifically focus on the potential mechanisms by which chronic stress could inhibit the efficacy of radiation therapy. We conclude that there is significant evidence for increased suppression of anti-tumor immune responses along with induction of tumor cell survival pathways. Because cancer patients are susceptible to many sources of stress, including stress associated with anxiety and depression, this survey provides a strong rationale for implementing stress-reduction strategies in patients who will be receiving radiation therapy.

**Abstract:**

Ionizing radiation has been used in the treatment of cancer for more than 100 years. While often very effective, there is still a great effort in place to improve the efficacy of radiation therapy for controlling the progression and recurrence of tumors. Recent research has revealed the close interaction between nerves and tumor progression, especially nerves of the autonomic nervous system that are activated by a variety of stressful stimuli including anxiety, pain, sleep loss or depression, each of which is likely to be increased in cancer patients. A growing literature now points to a negative effect of chronic stressful stimuli in tumor progression. In this review article, we present data on the potential for adrenergic stress to influence the efficacy of radiation and in particular, its potential to influence the anti-tumor immune response, and the frequency of an “abscopal effect” or the shrinkage of tumors which are outside an irradiated field. We conclude that chronic stress can be a major impediment to more effective radiation therapy through mechanisms involving immunosuppression and increased resistance to radiation-induced tumor cell death. Overall, these data highlight the potential value of stress reduction strategies to improve the outcome of radiation therapy. At the same time, objective biomarkers that can accurately and objectively reflect the degree of stress in patients over prolonged periods of time, and whether it is influencing immunosuppression and radiation resistance, are also critically needed.

## 1. Introduction

Chronic stress is a direct consequence of repeated exposure to emotional or physical pressures for a prolonged period of time, which can interfere with a person’s ability to handle normal circumstances in life and can sometimes be dangerous to health. Many people diagnosed with cancer naturally develop increased symptoms of chronic stress; unfortunately, mounting evidence from epidemiological and clinical studies have collectively demonstrated strong associations between chronic stress and cancer progression [1]. Consequently, there has been an increasing interest in deciphering and understanding the mechanisms underlying stress–cancer relationships. Chronic stress may even negatively influence the efficacy of cancer therapies. Radiation, which is the most common modality used for treating solid tumors, has been used for over 100 years. However, the ability of radiation to cure cancer completely is still not a reality, although the delivery technology and scientific underpinnings of radiotherapy have been improved.

In this review, we summarize how chronic stress and the secretion of various stress hormones may influence the outcome of treatment, in particular radiation therapy (RT), in cancer patients. We discuss the mutual effects of radiation and chronic stress on tumor microenvironments, as well as multiple factors contributing to radioresistance in cancer cells, including cell cycle, autophagy, senescence, cancer stem cells, neuroendocrine differentiation, hypoxia, metastasis, and metabolism. We also summarize some aspects of our current understanding of how chronic stress/adrenergic signaling could influence the effect of radiation on the anti-tumor immune response, and in particular, the function of cytotoxic immune cells and suppressive immune cells, respectively. We highlight new data exploring the underlying mechanisms by which treatment with blockers of β-adrenergic receptor signaling (i.e., β-blockers) increases the frequency of the “abscopal effect” following radiotherapy and related factors involved in triggering abscopal effect.

## 2. Chronic Stress and Cancer

Stress occurs frequently in everyone’s life. One type of stress results in what is known as the “fight-or-flight” reaction; it is associated with an activation of the autonomic nervous system resulting in an increase in heart rate, blood pressure, and breathing rate, which prepares the body to either stay and deal with a threat or to run away to safety. This type of stress is usually brief or acute and is generally protective of the individual. However, sometimes, certain types of stress can last a long time and this longer-term, or chronic, stress, is now thought to lead to many serious health issues, including mental health problems, cardiovascular disease, obesity, menstrual disorders, gastrointestinal problems, and even higher risk of some cancers. Chronic stress can affect emotions and cognition by its modulation of nervous, immune, endocrine, digestive, reproductive, musculoskeletal, cardiovascular, and respiratory systems across adolescence, adulthood, and aging [2,3]. Epidemiological findings support the premise that chronic psychological stress including stressful life experiences, anxiety, depression, insufficient social support perception, etc., is related to increased cancer risk, progression, and mortality [4,5,6,7,8]. For example, while a Japanese prospective study in over 100,000 participants did not find any association between short-term stress and cancer incidence, it revealed that men with higher perceived stress levels had an 11% greater risk of developing cancer than those with low stress levels, particularly in smokers, alcohol drinkers, obese subjects, and subjects without a family history of cancer [9]. A higher level of stress was also found to disturb neuro-immunological systems and the functions of the neuroendocrine axes, leading to changes in hormone levels in blood, which increased the risk of breast cancer [6,10]. Psychosocial stress was associated with an increased incidence of colorectal, lung, head and neck, hepatobiliary, esophagus [11], prostate [12], bladder, rectal, stomach [13], and lymphoid or hematopoietic cancers [4] in some recent studies. Encouragingly, however, combined psychological interventions have a positive impact in cancer patients via decreasing stress and improving quality of life, and further improving prognosis [14].

Feelings of depression and anxiety are common when patients are coping with cancer [15]. They may feel grief and sadness due to uncertain plans and future caused by cancer progression and factors such as financial concerns. Having increased chronic pain which results from the tumor itself or from surgery, treatments, and tests, can also make cancer patients feel tired, frustrated, sad, and even angry. Insomnia is another particular concern in cancer patients, with difficulty falling asleep and frequent, prolonged nighttime awakenings [16]. Whether these longer-term psychological stressors cause a limitation of the response to cancer treatments as well as quality of life and survival is still under investigation.

Chronic stress is also mediated by a variety of stress hormones including glucocorticoids and catecholamines. Glucocorticoids are regulated through the hypothalamic–pituitary–adrenal axis (HPA axis). They play an important role in circadian rhythm and mediation of adaptive responses to stress [17]. Chronic stress can lead to multiple forms of HPA axis dysregulation and alteration in cortisol secretion by the adrenal cortex. The prolonged elevation of cortisol in the blood level has been shown to suppress T-cell functions [18] and impair our memory and ability to learn [19]. Social support and stress reduction are associated with lower cortisol levels [20].

A number of studies have demonstrated that the chronic disruption of the circadian rhythm can inhibit the secretion of corticosterone [21,22,23,24]. A direct consequence is the promotion of tumor growth and metastasis with involvement of circadian genes, such as high expression of the Bmal1 gene and low expression of the Per1 gene as shown with liver metastasis [25], though the process is reversible by enhancing circadian clock function [26]. In this regard, nightshift work has been considered as a risk factor for breast [27] and prostate [28] cancers due to disruption of endocrine rhythms. In addition, cortisol may interact with catecholamines in a synergetic manner. For example, cortisol increased β-adrenergic receptors (β-ARs) density and potentiated the isoproterenol (ISO)-induced increase in cAMP accumulation in lung cancer cells [29].

In addition to increasing the production of the hormone cortisol, chronic stress is also associated with elevated levels of catecholamines (e.g., norepinephrine (NE) and epinephrine), which are secreted into the blood circulation by adrenal glands and sympathetic nerve endings of the sympathetic nervous system (SNS) to aggravate tumor progression. The effects of released catecholamines are mediated via α- or β-adrenergic receptors (ARs). Vascular α-ARs (α1 and α2), which are expressed in arterial resistance and venous capacitance vessels [30,31], regulate blood pressure by maintaining vascular tone through vascular smooth muscle contraction by coupling to the Gq or Gi subtypes of G proteins, respectively [32]. The effects of epinephrine and NE on innate and adaptive immune cells are predominantly mediated by β-ARs (β1, β2 and β3 [33]), which are critical for transferring the effects of SNS stimulation by stress into immune responses. NE stimulation of β-ARs activates cyclic AMP (cAMP)-protein kinase A (PKA) intracellular signaling pathway involved in cell survival, proliferation, differentiation, and maturation [34].

Research into targeting β-ARs has contributed to the invention and development of β-blockers. Propranolol was the first clinically useful nonselective β1-and β2-ARs blocker, invented by Sir James Black, who was awarded the Nobel Prize in Medicine in 1988 [35]. β-blockers have been traditionally considered “cardioprotective” [36], since β-AR signaling is fundamental in the relationship between psychological stress and cancer. Increasing pre-clinical evidence and clinical trials show that propranolol displays distinct anti-cancer effects in multiple cancer types, including leukaemia, breast, melanoma, ovarian, angiosarcoma, neuroblastoma, prostate, pancreatic, colorectal, head and neck, etc. [37], while other drugs in the same class, such as carvedilol and nebivolol, also have anti-cancer efficacy [38]. Propranolol has been repurposed as an anti-cancer agent due to its effects on cellular proliferation [39,40,41], migration [40], invasion [42,43], apoptosis [39,41], angiogenesis [44], treatment sensitization [41,45,46], as well as on the immune system [47,48,49,50,51,52].

## 3. Modulation of the Tumor Microenvironment by Chronic Stress: Impact on Radiotherapy

Radiotherapy is an important therapeutic tool used in cancer treatment; it is estimated that about half of all cancer patients will receive RT during their course of illness [53,54,55]. The main goal of RT is to deprive cancer cells of their multiplication (cell division) potential by depositing ionizing energy that causes genetic changes. To date, it remains the method of choice for treating the majority of solid cancers. While most research in the field of radiation biology has focused on determining the mechanism by which ionizing radiation can kill tumor cells, more recent research has focused on the impact of RT on components of the tumor microenvironment (TME) involving different non-malignant cell types.

Recently, investigations of psychological stress using animal models demonstrated that exposure to psychological stress after irradiation could compromise the efficacy of RT, which were especially pronounced under a prolonged and intensive stress [56]. The results published by Andersen et al. indicated that psychological stress inhibited cellular immune components involved in the regulation of tumor growth in cancer patients, including natural killer (NK) cell toxicity and T-cell responses [57]. More recently, using C57BL/6J mice bearing LLC lung tumors exposed to conspecific mice receiving inescapable foot shocks, Zhang et al. explored the molecular and cellular mechanisms by which psychological stress affects tumor growth and response to therapeutic radiation [58]. Their findings indicated that long-term psychological stress can enhance tumor growth and diminish the effectiveness of RT, possibly through the elevated secretion of corticosterone and higher expression of Wnt1, Drosha, and vimentin in LLC-1 cells. More recent data reveals that tumor growth itself may be driven by neurogenesis, or the recruitment of nerves by tumors as they grow. It has been shown that tumor-infiltrating nerve-derived molecules support tumor growth and dissemination [59]. Since the SNS can be continuously activated by chronic stress [60], the close proximity of these nerve endings to the tumor microenvironment raises considerable concern about the direct effects of secreted catecholamines within tumors [61]. Elevated secretion of NE by chronic stress has considerable potential to influence the tumor milieu, particularly those aspects which could be affecting the efficacy of radiotherapy.

### 3.1. Impact of Chronic Stress on Immune Cells

There has been a much greater appreciation of how much the efficacy of radiation actually depends upon the immune system. A recent meta-analysis by Segerstrom and Miller [62] based on effect sizes derived from 293 independent studies associated chronic stress with reduced NK cell cytotoxicity, suppressed lymphocyte proliferative responses and blunted humoral responses to immunization via β-ARs expressed on immune cells. Radiotherapy can lead to immunogenic cell death and the release of tumor antigens by irradiated tumor cells. These antigens are taken up by professional antigen-presenting cells such as dendritic cells. Upon maturation, the dendritic cells (DCs) migrate to the lymph nodes (LNs) where they present the processed antigens in the context of major histocompatibility complex (MHC) molecules to T-cells. Activation of CD8^+^ T-cells requires the cross-presentation of exogenous tumor antigens on MHC class I molecules. Naive CD8^+^ T-cells receive the antigen-specific signal through the T-cell receptor and co-stimulatory signals such as CD80 and CD86 through CD28. Tumor antigen-specific CD8^+^ T-cells proliferate and differentiate into cytotoxic effector T-cells that migrate from the LNs to the tumor sites (primary tumor and nonirradiated tumor metastases) in order to exercise their effect of killing tumor cells. However, cytotoxic T-lymphocyte-associated antigen 4 (CTLA-4) binds to CD28 competitively with CD80/86 and inhibits the activation of T-cells. Following T-cell activation, programmed cell death protein 1 (PD-1) receptors are expressed on the T-cell surface, which bind primarily to programmed death ligand 1 (PD-L1) and inhibit the activity of the CD8^+^ T-cells [63].

The release of radiation-induced cytokines in the tumor microenvironment influences the delicate balance between immune clearance and immune tolerance. For instance, the induction of interleukin-6 (IL-6), IL-10, and CSF-1 contributes to the proliferation and invasion of tumor cells and thereby displays a pro-tumorigenic role. In contrast, the secretion of pro-inflammatory IL-1β enhances the anti-tumor immune response. Furthermore, the differential expression of RT-induced chemokines determines the type of leukocyte infiltration in the tumor microenvironment. For example, the production of CXC-motif chemokine ligand 12 (CXCL12) results in chemotaxis of pro-tumorigenic CD11b^+^ myeloid-derived cells, whereas the upregulation of CXCL9, CXCL10, and CXCL16 can increase the expression of adhesion molecules such as E-selectin, intercellular adhesion molecule 1 (ICAM-1), and vascular cell adhesion protein 1 (VCAM-1) on endothelial cells and attract anti-tumor effector T-cells [64]. These effector T-cells migrate back to the tumor, attracted by chemokines released due to irradiation-induced cell destruction. In addition, these effector T-cells can induce tumor cell death in nonirradiated lesions distal to the initial irradiation site [65].

Radiation treatment induces interferons (IFNs), which not only directly enhance the immune response of dendritic cells (DCs) and CD8^+^ T-cells [66], but also contribute to radioresistance. Chen et al. found that intact type I IFN signaling in irradiated tumors promotes tumor growth post radiation and downregulation of *Serpinb9*, an IFN-inducible gene and inhibitor of granzyme B (GzmB) in the *Ifnar1*-knockout (KO) cancer cells, significantly reduces resistance to T-cell killing [67].

**Figure 1:**
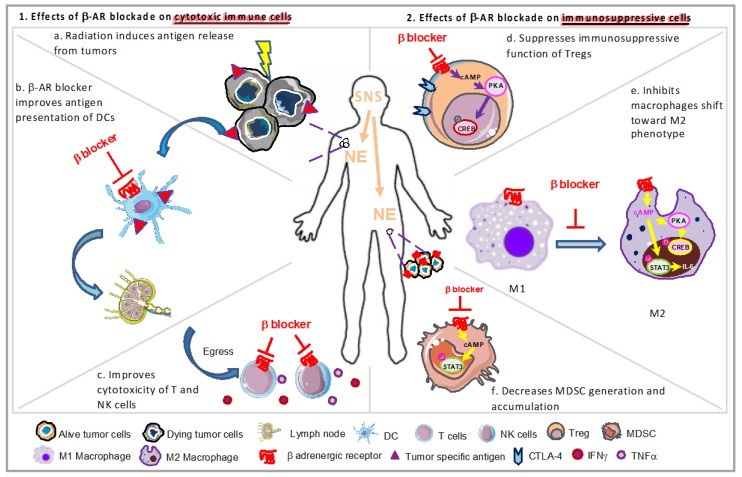
The improved efficacy of radiation therapy (RT) with β-AR blockade is induced by modulation of function of immune cells. After antigen release induced by radiation from tumor cells (**a**), β-blocker (**b**) improves antigen presentation of dendritic cells (DCs), (**c**) facilitates activation of T lymphocytes, their egress from lymph nodes (LNs), and the secretion of IFNγ and TNFα, as well as increases cytotoxic activity of natural killer (NK) cells. On the other hand, β-blocker (**d**) decreases CTLA-4 expression and suppresses the suppressive function of Tregs in a protein kinase A (PKA)-dependent way, (**e**) inhibits macrophages shift toward M2 phenotype via cAMP/PKA/CREB/IL-6/STAT3 pathways, and (**f**) decreases the generation and accumulation of myeloid-derived suppressor cells (MDSCs), dependent upon STAT3 phosphorylation.

Mounting epidemiological, preclinical, and clinical evidence supports that psychological factors, specifically chronic stress, promote tumor growth, progression, and metastasis via prolonged activation of the SNS and HPA axis and suppressed immunoprotection [1,68]. Some preclinical studies discovered that NE levels are higher, anti-tumor immunity is impaired, and tumor growth is accelerated by chronic (mild) cold stress in mice housed at standard room temperatures (ST, ∼22 °C). In this model, NE is increased because of its role in stimulating metabolic heat production. However, NE levels are reduced, immunosuppression is reversed, and tumor growth is slowed by housing mice at thermoneutral temperatures (TT, 30 °C) [50,69,70], suggesting a causative role for NE-mediated stress signaling in dampening antitumor immunity [71,72,73]. Elevated NE levels lead to dysregulation of adaptive and innate immune response, including decreased trafficking, infiltration, and function of antitumor CD8^+^ T-cells, increased T regulatory cell activity, impaired cytotoxicity of NK cells, repressed activation of DCs, accumulated myeloid-derived suppressor cells (MDSCs), and M2 macrophages in tumors, etc. [68,74]. For an illustration depicting the mechanistic overview of β-blockade application and impact on immune cells, see Figure 1. The detailed mechanisms are explained in the following section.

#### 3.1.1. Adaptive Immune Response

The primary function of the adaptive immune response is to distinguish what is *foreign* from what is *self*. The cells of the adaptive immune system include: antigen-specific T-cells, which are activated to trigger a rapid and efficient immune response through the action of antigen-presenting cells (APCs), and B cells which recognize antigens directly and differentiate into plasma cells to produce antibodies [75]. A tightly regulated interplay between these cells in adaptive immune systems facilitates pathogen-specific immunologic effector pathways, generation of immunologic memory, and regulation of host immune homeostasis [76].

##### CD8^+^ and CD4^+^ T-Cells

There is significant literature to support the idea that T-cell subsets mediate immune responses following radiation, and that they could be a target of stress. A recent study demonstrated in a melanoma model that tumor cells inhibit CD8^+^ T-cell infiltration after radiotherapy (30 Gy) and become radioresistant. Radiation impairs metabolic reprogramming of T-cell activation. Reduction of Myc expression post 3 Gy radiation compromises glucose transporter (Glut) expression and inhibits glucose uptake, glycolysis, and ATP generation in activated T-cells [77]. RT (12 Gy) causes macrophages to acquire an immunosuppressive M2 phenotype and suppresses T-cell mediated anti-tumor response in pancreatic tumor bearing mice [78]. High dose (>3.5 Gy) total body irradiation (TBI) leads to a massive killing of blood cells such as lymphocytes and even severe injury to hematopoietic stem cells including complete ablation of functions [79]. On the other hand, low dose (1.2 Gy) radiation in vitro alone can’t activate T-cells, but radiation combined with T-cell activation enhances proliferation and function of CD8^+^ and CD4^+^ T-cells including elevated IFNγ production, extracellular-signal-regulated kinase1/2 (ERK1/2), Akt phosphorylation, and glucose uptake [80]. These studies suggest that high-dose irradiation might hamper anti-cancer immunity.

Regarding the possible mechanism(s) by which stress can suppress the anti-tumor immune response following radiation, some intriguing data establish some testable hypotheses. Bucsek et al. [50] investigated the relationship between ambient temperature-induced stress and anti-cancer immunity and found that effector CD8^+^ T-cells were suppressed when the tumor bearing mice were exposed to mild chronic stress. They linked this effect to increased stress-induced β2-AR stimulation in CD8^+^ T-cells. A recent study [81] demonstrated that stressed tumor bearing mice had a poorer response to radiation; conversely, an improved anti-tumor response, and increased numbers of CD8^+^ and CD4^+^ T-cells expressing IFNγ and GzmB in irradiated tumors were found in the tumors of mice treated with propranolol, suggesting that β-AR signaling on lymphocytes might be involved in hampered immunity by irradiation. Altogether, the aforementioned studies reveal important mechanisms by which stress can block immune responses following radiation.

##### Regulatory T-Cells (Tregs)

Both human natural and TGF-β1-induced CD4^+^ Tregs are more resistant to 10 Gy radiation-induced cell death than CD4^+^ conventional T-cells [82]. Single or fractionated local irradiation increases Tregs in a radiation dose-dependent manner without affecting the functional integrity of Tregs [83]. Tregs are also reported to increase in the circulation in colorectal cancer patients following radiotherapy [84]. But another study reported that 10 Gy irradiation causes a long-term infiltration of intestine by function-impaired Tregs [85]. Activation of β2-AR in Treg cells by noradrenaline increases intracellular cAMP levels and PKA-dependent cAMP response element-binding protein (CREB) phosphorylation, leading to enhanced CTLA-4 expression and suppressive function of Treg cells [86]. Moreover, Zhou et al. found that surgical stress induced immunosuppression due to elevated levels of circulating epinephrine, NE, forkhead box P3 (Foxp3) mRNA, and Treg frequencies in breast cancer patients, while propranolol administration could attenuate such elevation of Tregs [87]. These studies indicate that radiation and adrenergic signaling contribute to an increased number and the immunosuppressive function of Tregs.

#### 3.1.2. Innate Immune Response

Innate immunity is essential to the onset and maintenance of adaptive immunity and fully integrates the cancer-immunity cycle [88], in which innate immune cells participate in all steps of T-cell generation and activity against cancer cells, by participating in tumor-specific T-cell priming, expansion and infiltration at the tumor site [89]. Innate immune cells, including macrophages, DCs, and NK cells, are key mediators of the radiation-induced immune response. RT induces both impaired tumor and normal cells to release multiple specific danger signals including hsp70, high mobility group box 1 (HMGB1), calreticulin, cytosolic deoxyribonucleic acid (DNA), complement, and ATP, which are sensed by innate immune cells such as macrophages or DCs via toll-like receptor 4 (TLR-4), cyclic GMP-AMP synthase (cGAS)-stimulator of interferon genes (STING), CD47 and nucleotide-binding domain leucine-rich repeat family pyrin domain containing protein 3 (NLRP3), and then trigger nuclear factor kappa B (NF-κB) and interferon regulatory factor 3 (IRF3) leading to downstream cytokines/chemokines production [90].

##### Dendritic Cells 

In a recent phase II randomized trial, preoperative β-blockade with propranolol was found to increase recruitment/activation of BDCA1^+^ classical myeloid DCs and BDCA2^+^ plasmacytoid DCs within the primary tumor of patients with breast cancer [91]. This strongly suggests that adrenergic signals can help regulate the function of dendritic cells. Since radiation is also thought to influence the function of dendritic cells, they represent an important target by which adrenergic stress could influence the efficacy of radiation. 

A low dose of TBI (0.2 Gy) was found to increase surface expression of CD80, CD86, MHC class I, and MHCII on immature and mature DCs, but suppressed the antigen uptake capacity as well as IL-12 secretion, suggesting a shift toward immune tolerance [92]. Mature DCs given a high dose of irradiation (30 Gy) secreted less IL-12, showed remarkable resistance against radiation-induced apoptosis and were less effective in a mixed lymphocyte reaction [93]. A recent study observed a significant decrease of BDCA3^+^ DC in the blood of patients treated with conventional irradiation (10 Gy). Moreover, consistently, CD8^+^ DC, (a mouse equivalent of human BDA3^+^ DC), also decreased in peripheral blood, spleen, and LNs post 6 Gy radiation, indicating a repression of Th1 immunity caused by irradiation [94].

DCs play an important role as the messengers between the innate and adaptive immune systems. When mature, they are excellent antigen-presenting cells which process and present antigen material on the cell surface to prime T-cells of the immune system. DCs can express α1-AR, α2-AR, β1-AR, and β2-AR. β2-AR, however, was found as the primary adrenergic receptor to modulate cytokine production and antigen presentation by DCs [95]. Depending on the differentiation and maturation status of DCs, the activation of β2-AR can be stimulatory or inhibitory to downstream signaling [95]. β2-AR activation on bone marrow DCs increases the differentiation of Foxp3 positive suppressive Treg cells [96]. β-AR agonist ISO inhibits DC maturation and promotes tolerance via inhibiting the translocation of NF-κB to the nucleus, thereby suppressing the expression of the costimulatory molecule CD86, MHCII and tumor necrosis factor alpha (TNFα), and promoting IL-10 secretion [97]. Another selective β2-AR agonist clenbuterol was also reported to inhibit the differentiation of human monocytes into DCs [98]. β2-AR signaling in DCs diminishes IL-12 secretion via inhibition of the NF-κB pathway, leading to low production of IFNγ but high secretion of IL-17, thus influencing adaptive immunity [99]. Thus, stimulation of β2-AR on DCs plays an immunosuppressive role in immune modulation due to a reduction of activated T-cells along with a decreased release of cytokine from DCs. β2-AR stimulation impairs phagosomal Ag degradation and inhibits NF-κB translocation to the nucleus in DC, which further suppresses upregulation of costimulatory molecules such as CD80, CD86 and CD40, decreases the immunostimulatory cytokines secretion such as IL-12, and inhibits Ag protein cross-presentation to CD8^+^ T-cells [100]. 

##### NK Cells

An in vitro study showed that low dose RT augmented expansion and cytotoxicity of cultured NK cells possibly through p38-mitogenic activated protein kinase (MAPK) pathway. However, McGinnes et al. found that cancer patients had decreased NK activity in peripheral blood after receiving radiotherapy to sites including the mediastinum [101]. Another study also reported that the levels of NK cells were significantly reduced in breast cancer patients treated with radiotherapy with/without chemotherapy [102]. These studies suggest that NK cells can influence the efficacy of radiation and therefore we include here some literature showing how stress influences NK cell activity.

Continuous stress by exposure to a wet cage or administration of a β-adrenergic agonist can disrupt the immunostimulatory efficacy of IL-2 on NK cell numbers and activity in rats [103]. Environmental stress (intermittent forced swim stress) and/or stress factor (administration of physiologically relevant doses of epinephrine) promote leukemia progression through diminished NK activity, which can be reversed by prolonged β-adrenergic blockade (nadolol) treatment [104]. Catecholamines from the adrenal glands activate β1- and β2-adrenoceptors and suppress NK cell activity and consequent host resistance to NK-sensitive metastasis [105]. NE can inhibit cytotoxicity and downregulate the expression of perforin, GzmB, IFN-γ of NK cells in a dose-dependent manner, mainly via the β2-AR/cAMP/PKA/p-CREB signaling pathway [106].

The number of NK cells in the lungs and blood of the mice exposed to restraint stress was decreased via activation of β-AR, which can be reversed by the administration of a pan β-AR blocker, propranolol [107]. Similar results were reported with another MADB106 mammary adenocarcinoma model using non-selective β-AR blocker nadolol [108]. These studies indicate that β-blockers normalize the stress-suppressed NK cell activities and peripheral distribution [109]. In vivo study demonstrated that β-adrenergic stimulation peripherally suppresses NK activity which can compromise host resistance to NK-sensitive tumors [110]. Plus, adrenergic suppression of NK activity might be modulated by oestrous cycle [111], age [112], and gender [113].

The observation that the function of NK cells is suppressed by stress hormone is also verified in clinical studies. Daughters of breast cancer patients who experienced high levels of distress exhibited increased concentrations of NE, along with lower secretion of IL-2, IL-12, and IFNγ, which is associated with decreased natural cytotoxic activity of NK cells [114]. The patients who gained enhanced psychosocial well-being post mindfulness-based stress reduction (MBSR) programs which can reduce anxiety and overall distress showed increased NK cytolytic activity and decreased levels of C-reactive protein [115]. This literature does indeed suggest that stress can negatively influence the outcome of RT through its effects on NK cell number and function. 

##### Tumor-Associated Macrophages

Macrophages are a type of immune cell with remarkable plasticity that can execute a wide spectrum of functions, ranging from modulating tissue homeostasis, defending against pathogens, and facilitating wound healing [116]. Macrophages infiltrating and residing in the microenvironment of solid tumor tissues are defined as tumor-associated macrophages (TAMs), and more than 50% of tumor-infiltrating cells are TAMs [117]. These TAMs can be activated by various signals in the tumor microenvironment to exhibit dramatic impact on tumor progression and metastasis, and studies have shown that cancer prognosis is inversely correlated with the number of TAMs [118]. Previous studies [119] have collectively indicated that radiation induces recruitment of TAMs within a tumor, resulting in the increased secretion of proangiogenic cytokines, the recovery of the vascular network, and accordingly, tumor regrowth. For example, high doses (25–60 Gy) of single or fractionated RT triggers macrophages shift toward the tumor-promoting M2-phenotype with high levels of Arg-1, cyclooxygenase-2 (COX-2), and inducible nitric oxide synthase (iNOS) [120].

Social isolation induced chronic stress by adrenergic receptor β2 activation promotes breast cancer progression through macrophages M2 polarization in tumor microenvironments [121]. Genome-wide transcriptional profiles showed β2-AR-stimulated macrophages located on the M2-side of the M1-M2 macrophage spectrum. These M2-promoting effects were associated with CREB, CCAAT-enhancer-binding protein β (C/EBPβ) [122], and activating transcription factor (ATF) pathways [123]. G protein-coupled receptor kinase 2 regulating β2-AR signaling activates M2-polarized macrophages via downstream cAMP/PKA/CREB/IL-6/STAT3 signaling pathways and promotes the secretion of pro-tumor cytokines, which contribute to the proliferation, migration, and invasion of hepatocellular carcinoma [124]. Some in vivo studies also demonstrated that propranolol could sharply reduce the number of M2 macrophages in chronic stress-induced breast cancer models [121,125]. Thus, macrophage function could be a significant target for adrenergic signaling and thus influence the radiation response.

##### Myeloid Derived Suppressor Cells (MDSCs)

MDSCs are known to inhibit antitumor T-cell responses and confer radioresistance through expression of high levels of Arginase-I [126]. In 2010, Kioi and colleagues found that the hypoxic tumor microenvironment of patients with glioblastoma multiforme post-irradiation resulted in higher levels of HIF-1 protein in tumors. In turn, the induction of HIF-1 promoted the mobilization of CD11b^+^ myelomonocytes from bone marrow into the tumor and restored the radiation-damaged vasculature by vasculogenesis, which allowed the surviving tumor to continue to thrive [127]. STING/type I IFN pathway activation drives extrinsic radioresistance and suppressive inflammation by recruiting CCR2^+^ MDSCs post 20 Gy radiation [128]. When the primary prostate tumor was treated with radiation (3 Gy × 5), systemic increased numbers of MDSCs were found in the spleen, lung, lymph nodes and peripheral blood while macrophage colony-stimulating factor 1 (CSF1) also increased in irradiated tumors, consistent with increased serum levels of CSF1 in patients after radiotherapy, which resulted from the recruitment of the DNA damage-induced kinase ABL1 into the cell nucleus, promoting CSF1 gene transcription. If a selective inhibitor of CSF1R was used combined with radiotherapy, tumor growth was suppressed post local radiation [129]. Besides, exposure to ionizing radiation induces NFκB and upregulates COX2 [130], elevating the production of prostaglandin E2 (PGE2), thereby potentiating the suppressive phenotype and function of MDSCs [131].

NE induced by psychological depression triggers the release of neuropeptide Y via activation of β2-AR on the prostate tumor cells and significantly increases numbers of MDSCs in the tumor and spleen [132]. Another study from our group recently demonstrated that β2-AR activation induced by chronic stress increases MDSC generation and accumulation and that this is dependent upon signal transducer and activator of transcription 3 (STAT3) phosphorylation and Fas–Fas ligand (FasL) interactions. The effects of stress on MDSCs in this study were found to be inhibited by the addition of propranolol [51].

In summary, the above discussion highlights research showing how adrenergic stress signaling can influence major cells involved with anti-tumor immunity. These mechanisms may help to explain why stress can affect the outcome of RT. In the next section we discuss data regarding the impact of stress on tumor cells themselves, thus potentially influencing intrinsic radiation sensitivity. 

### 3.2. Impact of Chronic Stress on Irradiated Tumors

One of the hallmarks of cancer cells is that they divide uncontrollably and maintain sustained proliferative signaling during cell division. The cell cycle phase is an important factor to consider during radiotherapy because radiation primarily kills the cells that are actively dividing; it does not work very effectively on cells that are in the resting stage (G0). The radiosensitivity/radioresistance is considered as a major determinant of tumor response to radiation [133]. Exposure to irradiation induces DNA damage with single strand breaks or double strand breaks and activates ataxia-telangiectasia mutated (ATM) protein kinase [134], which results in two biological outcomes, cell death or cell survival. Apoptosis or necrosis will occur depending on the dose of radiation and if the DNA damage is too extensive, it leads to irreversible cell death. In the cancer cells which survive the irradiation, DNA damage can arrest cell cycle permanently along with the expression of markers of cellular aging, making the cells undergo senescence. Moreover, the cell cycle is blocked to allow DNA repair in the surviving cancer cells. Altogether, alterations in these factors of biological outcomes can lead to the generation of radioresistance [135]. In addition, the maintenance of cancer stem cells, neuroendocrine differentiation, hypoxia, metabolism, and proliferation in cancer cells also contributes to radioresistance and tumor progression.

**Figure 2:**
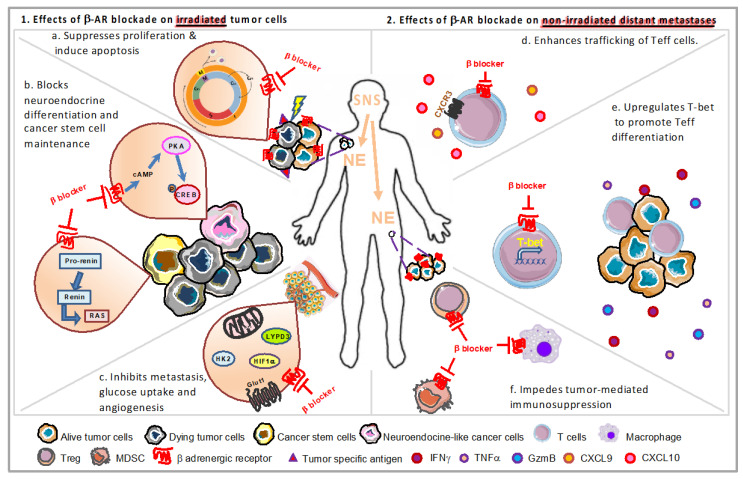
Administration of β-AR blockade can enhance the efficacy of RT by modulation of tumor microenvironment in irradiated and non-irradiated tumors. 1. For irradiated tumors: β-blocker (**a**) suppresses proliferation and induces apoptosis via modulation of cell cycle. (**b**) suppresses neuroendocrine differentiation by blocking cAMP/PKA/CREB pathway and inhibits the conversion of prorenin to renin and downregulates RAS which is required for cancer stem cell maintenance and differentiation. (**c**) inhibits HIF-1α mediated angiogenesis and LYPD3 induced metastasis, and mitochondrial and glucose metabolism in cancer cells. 2. For non-irradiated tumors: β-blocker (**d**) enhances trafficking of effector T-cells by upregulation of CXCR3 and its ligands CXCL9, CXCL10 to the non-irradiated tumor, (**e**) improves expression of IFNγ, TNFα, GzmB and T-bet, leading to infiltration of effector T-cells and effective elimination of tumor cells in the non-irradiated tumor, (**f**) decreases the number and immunosuppressive function of tumor-associated macrophages, MDSCs and regulatory T-cells, and effectively overcomes tumor immunosuppression.

β-ARs are expressed across diverse human cancer types. Rains et al. found that β1-AR and β2-AR were detected most highly in pancreas adenocarcinoma, melanoma and lung adenocarcinoma while β3-AR was detected most highly in melanoma [136]. Chronic stress can induce tumorigenesis and promote cancer development via aggravation of proliferation, invasion and metastasis in tumor cells through β-AR signaling [137]. Increasing evidence now shows that incidental β-blocker usage may improve the outcome of multiple cancers perhaps due to their ability to decrease the proliferative potential of cancer cells [138]. In the following sections, we will discuss the possible underlying mechanisms by which stress signaling modulates radioresistance in cancer cells (Figure 2).

#### 3.2.1. Impact of Stress Signaling on Tumor Proliferation/Apoptosis through Cell Cycle

Cell cycle proteins have been used as promising targets in cancer therapy [139]. In a study of breast cancer, the addition of propranolol was shown to reduce the percentage of the cell population residing in the G2/M phase of the cell cycle and increase the sub-G1 cell population, representing dead or dying cancer cells, along with altering the protein expression of the cell cycle regulatory proteins cyclin A, D1, E1, and E2. This suggests that blocking β-AR signaling can enhance cell death in late stages of breast cancer [140] (Figure 2a). Propranolol treatment also reduces the expression of pro-proliferative Ki67 in the early stage of breast cancer, decreases phosphorylation of p44/42 MAPK, p38 MAPK, c-Jun N-terminal kinase (JNK) and CREB in mitogenic signaling, elevates phosphorylation of cell survival/apoptosis regulators AKT, p53 and glycogen synthase kinase 3β (GSK3β) in SK-BR-3 breast cancer cell line, leading to induction of apoptosis and suppressed tumor proliferation [141].

The activation of the β-ARs can increase the proliferation of hemangioma-derived endothelial cells via upregulation of the ERK signaling cascade, which is effectively abolished by a β2-selective antagonist due to G0/G1 phase cell cycle arrest associated with decreased expression of cyclinD1, CDK-4, CDK-6, and phospho-Rb [142]. Similarly, propranolol also induces G0/G1/S phase arrest and apoptosis, leading to inhibition of proliferation in melanoma in vitro and in vivo by suppressing the AKT/MAPK signaling pathway [143]. Similar results were found in human ovarian cancer [144]. β2-AR blockage induces G1/S phase arrest and apoptosis in pancreatic cancer cells via Ras/Akt/NFκB pathway [145], or EFGR-Akt/ERK1/2 signaling pathway in colorectal cancer [146]. Activation of β2-AR signaling induces phosphorylation of PKA substrates CREB, vasodilator-stimulated phosphoprotein (VASP), Bcl-2 antagonist of cell death (BAD) and increases expression of myeloid cell leukemia 1 (MCL-1), leading to inhibition of apoptosis in prostate cancer [147]. In addition, RT induces expression of Ras related C3 botulinum toxin substrate 1 (RAC1), a member of the Rho family GTPase which promotes cell division via cell cycle [148], and enhances radioresistance via p21-activated kinase 1 (PAK1)-LIM kinase 1 (LIMK1)-Cofilins signaling in lung cancer [149]. Bachmann et al. found that β-AR can activate guanosine triphosphate (GTP)-Rac-bound PKA signaling pathway to the Raf–Mek–Erk cascade associated with cell cycle arrest in ovarian cancer cells. These cell-based studies revealed that β-AR-activated PKA phosphorylates the main Rac-effectors and p21-activated kinases, which leads to the elevation of downstream Erk1/2 signaling in a GTP-Rac1-dependent manner [150]. From these reports, we can see strong associations being established for the ability of β-AR signaling to contribute to tumor proliferation and survival by modulation of the cell cycle.

#### 3.2.2. Stress Hormones Can Induce Neuroendocrine Differentiation

Up to 60% of patients with advanced prostate cancer experience recurrence within five years after radiotherapy [151]. To explore the underlying mechanism of radioresistance and tumor recurrence, Deng et al. found that fractionated ionizing radiation induces differentiation of prostate cancer cells into neuroendocrine-like cells in vitro, in vivo and in prostate cancer patients [151] through activating CREB and impairing the nuclear import of ATF2, leading to prostate cancer progression and poor prognosis [152]. Increased activation of CREB was observed during the course of fractionated ionizing radiation-induced neuroendocrine differentiation that constitutes several distinct phases: (1) a fraction of cells selectively survive due to radioresistance although cell growth is largely inhibited during the first two weeks; (2) surviving cells differentiate into neuroendocrine-like cancer cells during the second two weeks; (3) continued irradiation cannot induce cell death of these differentiated cells during the last three weeks, which also can reverse to the proliferating state after the completion of the fractionated radiation treatment [153]. Neuroendocrine differentiation also can be induced by activation of CREB by intracellular cAMP/PKA signaling pathway [154] (Figure 2b). Neuroendocrine-like cells can promote tumor progression by the production of peptide hormones and growth factors and contribute to failure of radiation treatment due to reversal of the proliferating state [155]. Neuroendocrine differentiation also occurs in non-small cell lung cancer and contributes to radioresistance and increases metastatic potential via cAMP/CREB and IL-6-MEK/Erk signaling pathway [156]. A clinical study [157] also showed that colorectal carcinoma metastases contain a higher percentage of neuroendocrine differentiated cells as compared to their corresponding primary tumors.

Accumulating evidence shows that β2-AR signaling contributes to the progression and therapy resistance of prostate cancer via regulating trans-differentiation of cancer cells to neuroendocrine-like cells and thus affecting apoptosis, angiogenesis, epithelial-mesenchymal transition, migration, and metastasis. β2-AR is the most abundant receptor for sympathetic signals on prostate luminal cells [158]. Catecholamines, including epinephrine and NE which are secreted by sympathetic nerves in response to chronic stress can activate β2-AR. Upon ligand-binding, the expression of anti-apoptotic and pro-angiogenic factors is increased and a number of cancer cells undergo trans-differentiation to neuroendocrine-like cells and stimulate angiogenesis, neovascularization, and invasion contributing to prostate cancer progression and therapy resistance [158]. The use of β2-AR antagonist ICI 118,551 may overcome prostate cancer radioresistance [159].

#### 3.2.3. Cancer Stem Cells and Stress

Cancer stem cells, which initiate tumorigenesis and promote cancer cell migration, invasion, and metastasis, are considered critical for resistance and recurrence detected after radiotherapy. The survival of cancer stem cells post-radiation is known to be affected by cellular damage induced by radiation, the abscopal effect, and death receptor-ligand interaction [160]. The renin-angiotensin system (RAS) is demonstrated to be involved in cancer stem cell maintenance, function, and differentiation and plays a crucial role in tumor development [161]. Besides suppression of renin release, β-blockers inhibit the conversion of prorenin to renin [162]. Propranolol treatment suppressed plasma renin activity, reduced aldosterone secretion, and downregulated renin–angiotensin–aldosterone (RAA) axis components, angiotensin-converting enzyme and angiotensin II receptor-2 [163] to control infantile hemangioma [164,165]. Collectively, reducing adrenergic signaling by β-blocker can decrease survival of cancer stem cells via modulation of RAS (Figure 2b).

#### 3.2.4. Modulation of Hypoxia by Stress Signaling

Most free radicals generated by radiation are reactive oxygen species (ROS), which is the major contributor to DNA damage. Hypoxia induces hypoxia-inducible factor-1α (HIF-1α) accumulation via ROS mediated pathways and regulates multiple mechanisms contributing to hypoxia radioresistance [166] (Figure 2c). A recent study demonstrated that β-AR is involved in hypoxia sensing and is necessary for HIF-1α accumulation [167]. Chronic stress induces pancreatic tumor growth and angiogenesis by upregulated expression of matrix metallopeptidase-2 (MMP-2), MMP-9, and vascular endothelial growth factor (VEGF), mediated by a HIF-1α-dependent β-AR signaling pathway [168]. β-AR signaling in breast cancer facilitates G protein-coupled receptor kinase 2 (GRK2) phosphorylation and then increases human antigen R (HuR) nuclear export by inducing phosphorylation of HuR. The binding of cytosolic HuR protein to HIF-1α mRNA increases HIF-1α function as a transcription factor, which thereby enhances the transcription and secretion of VEGF-C angiogenic factor [169]. Moreover, β-AR agonists, especially the β2-AR agonist, activate epidermal growth factor receptor (EGFR) and then Akt and ERK1/2 in a PKA-dependent manner, which in turn upregulate HIF-1α in pancreatic cancer cells even under normoxic conditions [170]. In vitro experiments demonstrated that propranolol reduces the expression of HIF-1α in hemangioma cells in a dose- and time-dependent manner, mainly by acting on β2-AR. An in vivo study verified that propranolol could cause regression of infantile hemangiomas by suppressing VEGF and STAT3 signaling pathways in an HIF-1α-dependent manner [171]. Propranolol decreased cell viability, migration, and tubulogenesis in hemangiomas through the HIF-1α-mediated inhibition of VEGF-A and downregulation of phosphoinositide 3-kinase (PI3K)/Akt and p38/MAPK pathways [172]. In other words, treatment with β-blockers in cancer patients with chronic stress could help reduce hypoxia-induced radioresistence caused by the upregulation of HIF-1α.

#### 3.2.5. Chronic Stress Can Promote Metastasis

Metastasis often compromises the efficiency of RT. Su et al. found that a sublethal dose of radiation increases the metastatic potential of human cervical cancer cells by K-Ras/c-Raf/p38 signaling [173]. Likewise, β-adrenergic system is also known to contribute to cancer metastasis such as cellular proliferation and apoptosis and angiogenesis and vasculature normalization [174]. NE treatment increased the migration of breast tumor cells and upregulated pro-metastasis Ly6/PLAUR domain-containing protein 3 (LYPD3), which was abrogated by β2-AR antagonist ICI-118551, suggesting LYPD3 plays as a potential key mediator in β2-AR driven metastasis [175] (Figure 2c). In addition, NE drives PC-3 human prostate cancer cells metastasis both in vivo and in vitro [176], which is inhibited by β blocker propranolol, indicating that β-ARs are essential for NE’s impact on prostate adenocarcinoma metastasis.

#### 3.2.6. Modulation of Metabolism in Cancer by Stress Hormones

Metabolic changes, including alterations in the glycolytic and mitochondrial metabolism, can induce radioresistance [177] and these interactions may be influenced by stress. For example, chronic stress-induced epinephrine can elevate glycolytic activator lactate dehydrogenase A(LDHA) via β2-AR, leading to glucose metabolic rewiring, and the adjusted pH directed USP28-mediated deubiquitination and stabilization of MYC, activating the SLUG promoter, thereby promoting development of breast cancer stem-like properties [178]. Upregulation of hexokinase 2 (HK2), a key glycolytic enzyme in the first essential step of glucose metabolism, can induce glycolysis, crucial for tumor progression. Inhibition of HK2 signaling pathways is considered to enhance radiosensitivity [179]. Propranolol was demonstrated to prevent glucose metabolism of 4T1 breast cancer tumors via downregulation of HK2 [180] (Figure 2c). In addition, propranolol was shown in a recent study to prevent prostate cancer progression by sensitizing cancer cells to glucose metabolism inhibition. Treatment of cancer cells with propranolol in combination with the glycolysis inhibitor 2DG induced a massive accumulation of autophagosome due to autophagy blockade, and efficiently prevents prostate cancer cell proliferation, induces cell apoptosis, alters mitochondrial morphology, inhibits mitochondrial bioenergetics and aggravates ER stress in vitro, and also suppresses tumor growth in vivo [41].

Hyperactive mitochondrial metabolic profile in recurrent/metastatic human papillomavirus-associated head and neck squamous cell carcinoma model is dependent on β2-AR expression [181]. A recent study verified that propranolol slows primary tumor growth, inhibits metastatic development with a significant reduction in tumor cell mitochondrial metabolism in a murine model of recurrent/metastatic human papillomavirus-associated head and neck squamous cell carcinoma [181]. Moreover, the combination of propranolol and dichloroacetate, a clinically available glycolytic inhibitor, sensitizes resistant cells to radiation [182]. Propranolol also achieves a complete suppression of the mitochondrial bioenergetics in breast cancer cells combined with low doses of metformin [183].

## 4. Can Stress Affect the Frequency of the “Abscopal Effect” following Radiation?

Radiotherapy often does not completely cure solid tumors and recurrences at the irradiated site or in sites outside of the treated field often occur in cancer patients following locoregional RT [184]. It has even been suggested that radiation can facilitate tumor recurrence through attraction of migrating tumor cells [185]. On the other hand, radiation can also result in the shrinkage or even disappearance of tumors that were not in the irradiated field. This is referred to as the “abscopal effect.” In 1953, RH Mole, a physician, proposed the term “abscopal effect” from the Latin “ab” (away from) and “scopus” (target), to refer to effects of irradiation “at a distance from the irradiated volume but within the same organism” [186]. Unfortunately, the occurrence of abscopal effects in the radiation oncology clinic is rare.

### 4.1. Emerging Evidence of a Role for Stress in Regulating the Frequency of the Abscopal Effect

Accumulating research shows that the immune system is a major determinant factor in modulating the abscopal effect [187]. For example, when breast tumor bearing mice were treated with radiation and anti-TGFβ, DC activation and CD8^+^ T-cell responses were effectively enhanced to regress irradiated tumors and nonirradiated lung metastases, namely, abscopal effect [188]. 

The infrequency of the abscopal effect in the clinical setting is likely due to the counterbalance of the pro-immunogenic signals generated by RT with the immunosuppressive effects of RT. RT promotes TGF-β levels, recruitment of MDSCs, and enrichment of regulatory T-cells, which play an immunosuppressive role [189,190]. Many studies have reported the recruitment of myeloid-derived cells, which are primarily M2 TAMs and MDSCs and usually promote tumor growth and immune evasion, as well as Tregs [64], after RT in the tumor microenvironment [190], which is associated with poor prognosis in cancer patients.

A recent study from our group demonstrated that the frequency of abscopal effect following radiation is highly dependent upon the degree of adrenergic stress in tumor-bearing mice [49]. In this study, using three different strategies to manipulate adrenergic stress, including physiological, pharmacological, and genetic strategies, major improvements were observed in the control of both irradiated and non-irradiated distant tumors in colon tumor and melanoma models when adrenergic stress or signaling through β2-AR is reduced. Further cellular and molecular evidence also indicated that enhanced effector function of antigen-specific CD8^+^ T-cells and T-cell egress from LNs play an important role in the absence of adrenergic signaling. The enhanced expression of some important effector molecules including IFNγ, TNFα, GzmB, and T-bet as well as the upregulation of CXCR3 on CD8^+^ T-cells was detected in non-irradiated tumors from β2-AR KO mice (Figure 2d,e). Even though the suppressed activation of CD8^+^ T-cells is recovered by blocking adrenergic signaling via β blockade [191], most effector lymphocytes must exit the LN to perform their immune functions [192]. However, β2-AR-mediated signals inhibited LN egress of antigen-primed T-cells, and reduced their recruitment into peripheral tissues [193,194].

Although the induction of anti-tumor T-cell responses, including upregulation of IFNγ and GzmB on CD8^+^ T-cells, in irradiated tumors has been detected [81], the proper trafficking of effector T-cells into the tumor microenvironment may not always occur [195]. The chemokine receptors expressed by activated effector T-cells suggest candidate chemokines that could favor their migration into tumor sites. IFN-γ-producing CD8^+^ T-cells express CCR5 (which can bind RANTES (regulated on activation, normal T-cell expressed and secreted), macrophage inflammatory protein 1α (MIP-1α), and MIP-1β), and CXCR3 (which can bind CXCL9, 10, and 11) [192]. Accordingly, the levels of IFNγ, TNFα and CXCL9 in serum also increased in these mice. On the contrary, immunosuppressive cells, i.e., M2 macrophage and Tregs (M. Chen, unpublished data), significantly decreased (Figure 2f). Thus, the efficacy of radiotherapy is likely to be compromised due to the existence of adrenergic stress in the tumor microenvironment, which suppresses the activation, effector function and trafficking of cytotoxic CD8^+^ T-cells, as well as the recruitment of immunosuppressive cells. Once the pro-tumorigenic role of adrenergic signaling is inhibited by β blockade, the activation of CD8^+^ T-cells is recovered [191]. Activated effector T-cells can then exit the LNs and migrate to tumors to induce cytotoxic effects on tumor cells. Meanwhile, recruitment, survival, and function of MDSCs and Tregs are also suppressed [50,51,196] (Figure 2f). This might be the possible reason why the study showed that combining RT with β blockade can effectively overcome tumor immunosuppression and trigger abscopal events compared to radiation alone. Furthermore, propranolol treatment also significantly improves radiation efficacy, especially the abscopal effect, in the irradiated tumor-bearing mice treated with anti PD-1 [49]. These data suggest that blockade of β2 adrenergic signaling could be a useful strategy to improve radiotherapeutic efficacy in cancer patients.

As mentioned above, reducing chronic stress might be useful for improving the frequency of the abscopal effect since we have shown that stress can influence not only the intrinsic radiation sensitivity of tumor cells, but also the indirect effect of radiation on the anti-tumor immune response. Interestingly, some clinical studies have started to explore the effect of stress reduction on the efficacy of radiotherapy. Clinical trial NCT00057733 is designed to determine whether stress management techniques such as muscle relaxation, guided imagery, and abdominal breathing may improve quality of life and decrease emotional distress in patients who are undergoing RT for cancer. Clinical trial NCT03538223 is to verify whether the perception of anxiety and stress in breast cancer patients undergoing radiotherapy is influenced by listening to music. A pilot study (NCT03728205) is being conducted on the effect of Yoga on fatigue and stress levels in solid tumor cancer patients undergoing active RT.

### 4.2. Variables That May Influence the Impact of Stress on the Abscopal Effect

There are several other important variables which may influence the abscopal effect and how it may be influenced by stress. For example, the size of the irradiated target volume may play in a role in determining the degree of an abscopal response. Larger tumors have been hypothesized to release a larger number and variety of neoantigens upon irradiation [197]. However, larger tumors may also shelter deeper hypoxic areas that are immunosuppressive and radioresistant [189]. Even the precise definition of the abscopal response varies in different clinical reports and preclinical studies. No consensus definition of abscopal effect exists [198]. In one study, the abscopal effect was defined as a 30% reduction in the size of one nonirradiated metastasis [199], while it was defined as the sum of the largest diameter for all the nonirradiated target metastases using Response Evaluation Criteria in Solid Tumors (RECIST) techniques in another report [200]. This variable is important since assessment of the impact of stress on the abscopal effect will require quantitative data.

The dose and fractionation also seem to impact the immunotherapeutic potential and the presence of an abscopal effect [201], and the optimal scheme remains controversial [201]. At radiation doses <0.5 Gy, generally too low to directly induce cell death, irradiated cells release oxygen and nitrogen radicals that activate innate immune cells, such as macrophages, to release cytokines. Depending on the environment and genetic background, this process can result in chronic inflammation that causes genetic alterations and cell death as a secondary event. It is in this setting that the immune-modulating effects of radiation promote mostly a pro-tumorigenic role of the immune system. Conversely, at doses sufficient to directly provoke significant cell death, radiation induces specific signals of danger that are sensed by innate immune cells such as dendritic cells, and lead to the activation of an adaptive immune response. In the case of cancer radiotherapy, this process can promote anti-tumor immunity. Therefore, the immune-modulating effects of radiation are influenced by the dose, the type of signals generated by irradiated and non-irradiated cells, and by the activation of different types of innate immune cells [199]. A recent analysis from a randomized study showed that mean white blood cell counts in prostate cancer patients were lower in the conventionally fractionated (five times per week) than moderately hypofractionated (four times per week) radiotherapy, suggesting a difference in leukotoxicity between fractionation schemes [202]. Ablative radiation (15–25 Gy × 1) generates strong immunity in CD8^+^ T-cell dependent manner by increasing T-cell priming in draining lymphoid tissues, which results in the reduction of primary tumor and eradication of distant metastasis [203]. Another study also showed that a single radiation dose (2–20 Gy) induced autophagy and MHC-I expression to increase CD8^+^ T-cell infiltration in non-small cell lung cancer cells [204]. Low-dose fractionated radiotherapy (2 Gy × 5) induced T-cell infiltration at the irradiated tumors and both tumor-resident and infiltrating T-cells contributed to the suppression of local and distant tumors when radiation was combined with PD-1 blockade [205].

The dose per fraction of radiotherapy seems to have an important impact on systemic consequences [63]. Considering that lymphocytes are exquisitely sensitive to radiation, repetitive daily radiotherapy can deplete migrating immune effector cells. Notably, the study of Vanpouille-Box et al. demonstrated that a radiation dose above a threshold of 10–12 Gy per fraction could attenuate the immunogenicity of cancer cells because of the induced upregulation of the DNA exonuclease three-prime repair exonuclease 1 [206]. However, the clinical data from patients regarding the abscopal effect shows that the RT doses and fractionation used varies widely in the patients. Fractionation schemes were evaluated in four different groups, including conventional fractionation (1.8–2 Gy/fr), moderate hypofractionation (3–6 Gy/fr), hypofractionation (7–10 Gy/fr), and ablative doses (>12 Gy). It was observed that doses of 3–5 Gy/fr are the preferred scheme for inducing abscopal effect [198].

The data from a recent review showed that in most of the reports evaluated (23 out of 24), the abscopal response occurred in patients who received RT concurrent with, or immediately after immunotherapy, suggesting that the immunotherapy should be applied prior to/or concomitant use of radiation [198]. Other clinical reports from cancer patients also showed that the abscopal effect occurred in patients with melanoma, breast cancer or non-small lung cancer, who received RT concurrent with or post immunotherapy including a DC vaccine, TGF-β, PD-1, or CTLA-4 blockade treatment [63,64,207,208,209,210,211,212,213]. Preclinical models support the use of prior and/or concomitant administration of immunotherapy more than RT followed by immunotherapy [214]. Thus, for β blockade, the optimal dose and sequencing also needs to be considered in combination with radiotherapy based on both preclinical data [49,215], and clinical studies [216,217,218].

## 5. Health-Related Quality of Life Depends upon Psychosocial Stress and Has the Potential to Influence Outcomes Following RT

Most of the information reviewed here deals with molecular and/or immunological interactions which are likely to be influenced by stress and which are important in dictating the overall response to radiation. However, it is also important to remember that a crucial factor affecting tumor growth and the efficacy of radiotherapy is also likely to be the degree of stress, potential interactions among stressors, an individual’s personality, the available psychosocial support, and the effect of these interactions on an individual’s ability to cope with stress [219]. Cancer patients often experience a common set of stressors including anxiety, adjustment disorders, and depression, prior to, during, and after a course of radiation treatment. Psychological or behavioral interventions would be necessary to benefit quality of life [220,221]. The expected consequence of constraining the immune system in stressed patients with cancer would be diminished responses to radiation, and even shortened survival. Indeed, health-related quality of life (HRQOL) designed to characterize the patient experience of disease and treatment sequalae does have prognostic implications in a wide variety of tumors [222,223,224,225,226,227,228,229,230,231,232,233]. 

The complexity of understanding the significance of the interaction of various stressors is highlighted by the data which is emerging regarding patients with “financial toxicity” (FT). FT is defined as those “problems a patient has related to the cost of medical care” by the National Cancer Institute [234]. FT is thought to induce significant stress. Moreover, this stress may also worsen and prolong the stress resulting from the cancer diagnosis itself. Ramsey et al. linked regional cancer registry and federal bankruptcy records and found the adjusted mortality among patients with various cancers who filed for bankruptcy nearly doubled [235]. Our group investigated the effect of FT (level of worry) prior to chemoradiation treatment for head and neck cancer on survival. We also found that those who answered “somewhat” or “very much” compared to “not at all” or “a little” to the question “Has your physical condition or medical treatment caused you financial difficulties?” [236] had nearly double the risk of death [237]. This association only strengthened after propensity score match pairing. Using the same question, Klein et al. found that increasing baseline FT was significantly associated with shorter progression free survival (*p* = 0.01) in patients treated with chemoradiotherapy for locally advanced non-small-cell lung cancer [238]. Some have suggested FT at baseline should itself become a target for mitigation in prospective trials and clinical practice [239,240,241,242].

But in addition to quality of life assessment for the degree of stress a patient may feel, there is also a critical need for accurate and objective biomarkers of stress that could be assessed in samples that could be repeatedly obtained (e.g., from blood, urine, or saliva) during cancer treatment and survivorship. This could be used in combination with standardized QOL assessments, to provide the most accurate assessment of how much stress an individual is enduring and whether it is capable of influencing overall survival or efficacy of therapies such as radiation. This could take the form of measurement of catecholamine levels or as other, so far undefined markers of stress-induced immunosuppression. In summary, a variety of stressors, and their interactions should be taken into consideration when considering their impact on the efficacy of RT and whether stress reduction and/or β-AR antagonists should be used.

## 6. Conclusions

This review reveals that there is now considerable evidence that chronic stress has the potential to reduce the overall efficacy of ionizing RT against tumors. This evidence comes from pre-clinical models as well as clinical trials and population studies. The literature, as well as our own research, indicates that this depression of responsiveness to RT can occur through both impaired anti-tumor immunity, as well as an enhancement of intrinsic tumor cell survival mechanisms following radiation. Here we have included literature supporting both of these potentially overlapping mechanisms.

In terms of impact on anti-tumor immunity, chronic stress has been linked to impaired maturation and differentiation of DCs and their antigen presentation to T lymphocytes. β-AR stimulation induced by chronic stress also suppresses the cytotoxic function, trafficking and infiltration of effector T-cells and NK cells. On the other hand, stress and β adrenergic signaling results in an increased frequency of MDSCs, M2 macrophages and Tregs in the tumors, contributing to an immunosuppressive tumor microenvironment. In a very recent study [243], members of our team conducted the first Phase 1 clinical trial testing the combination of propranolol with Pembrolizumab and obtained very encouraging results that have led to a Phase II trial. This study supports the feasibility of combining drugs such as propranolol to block β-AR signaling and thereby improve immunotherapy. New trials combining propranolol with radiation are strongly warranted.

In addition to the effects on anti-tumor immunity, radioresistence is a major determinant of tumor response to irradiation. β-ARs are expressed in multiple human cancer types. Stimulation of β-ARs by chronic stress promotes proliferation, angiogenesis, neuroendocrine differentiation, metabolism, and metastasis of cancer cells, as well as maintaining function and differentiation of cancer stem cells, leading to the generation of radioresistance and tumor progression. Chronic stress and β adrenergic signaling may therefore be a major factor limiting the efficacy of radiotherapy, and reducing the frequency of the abscopal effect. Therefore, stress reduction strategies or β-AR blockade may have a two-prong benefit to improving RT-reducing immunosuppression and reducing radioresistance.

To improve the frequency of abscopal effects following RT, more preclinical research and clinical trials are needed to identify the maximal dose and timing of β-AR blockade to use as well as the optimal schedule of radiotherapy. Naturally occurring differences in the radiosensitivity of various tumor types and the feasibility of correlative endpoints (time of sampling and frequency) should be also carefully considered.

Although increasing evidence shows the frequency of abscopal effect can be improved by reducing stress signaling, the mechanism is still unclear. In the future, new research should focus on determining the underlying molecular pathways modulated by chronic stress in the network between immune cells and cancer cells, and the effect of chronic stress on bone marrow stem cells and precursor cells. Further, there is a great need to increase our understanding of intracellular pathways in tumor cells that can be influenced by β-AR signaling so that we can better understand how stress could contribute to intrinsic radioresistance among various tumor types. At the same time, interesting questions exist regarding the comparative benefit of psychosocial or behavioral stress reduction plans (e.g., exercise) vs. the use of pharmacological agents such as β-blockers. Moreover, the interactions of complex stressors with each other, such as anxiety following a cancer diagnosis combined with subsequent financial worries, may worsen and prolong stress effects in the tumor microenvironment. Together, these questions highlight a critical need for biomarkers which could objectively identify patients most in need of stress reduction efforts and/or pharmacological approaches over time which could be used in addition to the more subjective quality of life assessments.

In summary, while many questions remain to be addressed, it is clear that reducing chronic stress or the use of β-AR blockades could be an exciting new therapeutic strategy to reduce immunosuppression and increase radiosensitivity and thus be of great benefit to patients who will receive radiotherapy for the treatment of cancer.

## Abbreviations

ATF	activating transcription factor
ATM APC	ataxia-telangiectasia mutated antigen-presenting cells
BAD	Bcl-2 antagonist of cell death
β-ARs	β-adrenergic receptors
cAMP	cyclic adenosine monophosphate
C/EBPβ	CCAAT-enhancer-binding protein β
cGAS	cyclic GMP-AMP synthase
COX-2	cyclooxygenase-2
CREB	cAMP response element-binding protein
CSF1	colony-stimulating factor 1
CTLA-4	cytotoxic T-lymphocyte-associated antigen 4
CXCL12	CXC-motif chemokine ligand 12
DCs	dendritic cells
DNA	deoxyribonucleic acid
EGFR	epidermal growth factor receptor
ERK	extracellular-signal-regulated kinase
FasL	Fas-Fas ligand
Foxp3	forkhead box P3
FT	financial toxicity
Glut	glucose transporter
GRK2	G protein-coupled receptor kinase 2
GSK3β	glycogen synthase kinase 3β
GTP	guanosine triphosphate
GzmB	granzyme B
HIF-1α	hypoxia-inducible factor-1α
HK2	hexokinase 2
HMGB1	high mobility group box 1
HPA axis	hypothalamic-pituitary-adrenal axis
HRQOL	health-related quality of life
HuR	human antigen R
ICAM-1	intercellular adhesion molecule 1
IFNs	interferons
IL-6	interleukin-6
iNOS	inducible nitric oxide synthase
IRF3	interferon regulatory factor 3
ISO	isoproterenol
JNK	c-Jun N-terminal kinase
KO	knockout
LDHA	lactate dehydrogenase A
LIMK1	LIM kinase 1
LNs	lymph nodes
LYPD3	Ly6/PLAUR domain-containing protein 3
MAPK	mitogenic activated protein kinase
MBSR	mindfulness-based stress reduction
MCL-1	myeloid cell leukemia 1
MDSCs	myeloid-derived suppressor cells
MHC	major histocompatibility complex
MIP-1α	macrophage inflammatory protein 1α
MMP-2	matrix metallopeptidase-2
NE	norepinephrine
NF-κB	nuclear factor kappa B
NK	natural killer
NLRP3	nucleotide-binding domain leucine-rich repeat family pyrin domain containing protein 3
PAK1	p21-activated kinase 1
PD-1	programmed cell death protein 1
PD-L1	programmed death ligand 1
PGE2	prostaglandin E2
PI3K	phosphoinositide 3-kinase
PKA	cAMP-protein kinase A
RAA	renin-angiotensin-aldosterone
RAC1	Ras related C3 botulinum toxin substrate 1
RANTES	regulated on activation, normal T-cell expressed and secreted
RAS	Renin-angiotensin system
ROS	reactive oxygen species
RT	radiation therapy
SNS	sympathetic nervous system
ST	standard room temperatures
STAT3	signal transducer and activator of transcription 3
STING	stimulator of interferon genes
TAMs	tumor-associated macrophages
TBI	total body irradiation
TLR-4	toll-like receptor 4
TME	tumor microenvironment
TNFα	tumor necrosis factor alpha
Tregs	regulatory T-cells
TT	thermoneutral temperatures
VASP	vasodilator-stimulated phosphoprotein
VCAM-1	vascular cell adhesion protein 1
VEGF	vascular endothelial growth factor
